# IoT-Based Home Monitoring: Supporting Practitioners’ Assessment by Behavioral Analysis

**DOI:** 10.3390/s19143238

**Published:** 2019-07-23

**Authors:** Niccolò Mora, Ferdinando Grossi, Dario Russo, Paolo Barsocchi, Rui Hu, Thomas Brunschwiler, Bruno Michel, Francesca Cocchi, Enrico Montanari, Stefano Nunziata, Guido Matrella, Paolo Ciampolini

**Affiliations:** 1 Università degli Studi di Parma, Dip. Ingegneria e Architettura, Parco Area delle Scienze 181/A, 43124 Parma (PR), Italy; 2 WiMonitor S.r.l, Via G. Tacchi 1, 38068 Rovereto (TN), Italy; 3 National Research Council of Italy, Via Moruzzi 1, 56124 Pisa (PI), Italy; 4 IBM Research - Zurich, Saeumerstrasse 4, CH-8803 Rueschlikon, Switzerland; 5 Azienda Unita’ Sanitaria Locale Di Parma, Str. Quartiere, 2/A, 43125 Parma (PR), Italy; 6 Lepida S.c.p.A, Via del Borgo di S. Pietro, 90/c, 40126 Bologna (BO), Italy

**Keywords:** IoT, smart home, behavioural analysis, active assisted living (AAL), anomaly detection, continuous monitoring

## Abstract

This paper introduces technical solutions devised to support the Deployment Site - Regione Emilia Romagna (DS-RER) of the ACTIVAGE project. The ACTIVAGE project aims at promoting IoT (Internet of Things)-based solutions for Active and Healthy ageing. DS-RER focuses on improving continuity of care for older adults (65+) suffering from aftereffects of a stroke event. A Wireless Sensor Kit based on Wi-Fi connectivity was suitably engineered and realized to monitor behavioral aspects, possibly relevant to health and wellbeing assessment. This includes bed/rests patterns, toilet usage, room presence and many others. Besides hardware design and validation, cloud-based analytics services are introduced, suitable for automatic extraction of relevant information (trends and anomalies) from raw sensor data streams. The approach is general and applicable to a wider range of use cases; however, for readability’s sake, two simple cases are analyzed, related to bed and toilet usage patterns. In particular, a regression framework is introduced, suitable for detecting trends (long and short-term) and labeling anomalies. A methodology for assessing multi-modal daily behavioral profiles is introduced, based on unsupervised clustering techniques. The proposed framework has been successfully deployed at several real-users’ homes, allowing for its functional validation. Clinical effectiveness will be assessed instead through a Randomized Control Trial study, currently being carried out.

## 1. Introduction

The recent increase in life expectancy is a result of continuous improvements of quality of life in modern industrialized countries. However, due to a simultaneous decrease in birthrate, a new trend of progressive population ageing has emerged in such countries [1]. It is expected that, by 2020, almost 25% of the EU population will be over 60 years, and, by 2060, such percentage is projected to increase up to 30% [2]. Population ageing severely challenges social- and health-care systems, with ICT (Information and Communication Technologies) being expected to contribute to foster effective solutions. Active and Ambient Assisted Living (AAL, [3]) systems emerged to address these issues, aiming at making home environments more intelligent, and promoting a sustainable model of independent, augmented living. In fact, AAL techniques come in many flavors, ranging from assistive-oriented solutions up to prevention-oriented ones. In the former case, an AAL system can help in making services more accessible, by compensating physical or sensory impairments with new smart devices. For example, Brain–Computer Interfaces have been integrated within AAL ecosystems to allow severely motor-impaired users to achieve communication and home control [4,5,6], exploiting low-cost and easily deployable solutions [7,8]. On the other hand, AAL systems can also play an important role in prevention and active monitoring, especially in the realm of smart homes. Indeed, smart-home technologies are devoting progressively more effort in monitoring habits and behavioral patterns [9,10]: many automation functions can be optimized with the use of data, such as the activation of specific home functions, aimed at comfort or energy-management purposes. Nonetheless, smart home data can be also used to extract information, albeit indirectly, about inhabitants’ general wellbeing [11,12]. Very simple home sensors can be used for this purpose, posing no burden on the end-user, allowing for unobtrusive and continuous monitoring [13]. Behavioral analysis, indeed, may be regarded as an effective complement to more conventional health monitoring techniques, such as telemedicine.

This paper presents methodologies and results for the ACTIVAGE project [14], funded by the European Union Horizon 2020 programme; more specifically, the Italian Deployment Site (DS-RER) [15] is presented, based in the Emilia-Romagna Region (RER), aiming at introducing ICT-enabled services into the current practice of regional Health Service (AUSL, Local Health Authority). General practitioners, formal caregivers, care and case-managers are involved in the experimentation, creating an IoT-augmented care continuum network. The DS-RER approach is actually suitable for a wide range of use cases; however, in the pilot at hand, the target population has been narrowed to older adults (65+) recovering from a stroke event, and still suffering from its after-effects. Such a use-case is, indeed, numerically relevant: in Italy, stroke is the third cause of death (approximately, 10–12% of all deaths per year [16], after cardiovascular diseases and neoplasms); furthermore, about 7000 stroke adverse events are recorded each year in the Emilia-Romagna region [17]. The DS-RER approach aims at empowering care professional with additional information that is not commonly provided in current care practice, namely their behavioral routines. Such information can not be directly mapped to conventional clinical markers, for which telemedicine solutions already exist and are commonly used (e.g., blood pressure monitors, pulse oximeters and weight scales, among others); nonetheless, changes in behavioral habits may reflect changes in overall wellbeing of a person (e.g., sleep routines, toilet usage changes). Such behavioral information can be acquired in a continuous fashion, without any explicit user involvement (i.e., no compliance is required, in contrast to self-administered measurements). This effectively provides contextual information which, coupled to standard care practice, may help in providing a wider overview of subjects’ wellbeing and recovery process. The main contribution of this paper are twofold: first, technical and architectural solutions (involving hardware design and software/cloud services) are presented, specifically developed to enable behavioral data acquisition and safe storage. Then, methodologies for data analysis are presented; in particular, two case studies are reported, for conciseness’ sake: daily rests and toilet usage. Nonetheless, such methods can be straightforwardly adapted to many different analysis on data and behaviors gathered by the home sensors. Analysis of real-life data allowed to validate the system in both architectural and software components.

The rest of the paper is organized as follows. Section 2 presents methodologies for the project, discussing the choice of two DS-RER services to be analyzed, providing details on the ACTIVAGE study design and evaluation, as well as introducing the IoT sensor kit technology, the supporting cloud environment and the data analytics components. Section 3 presents the results. In particular, the critical issue of sensor power consumption is discussed. An example of daily rest pattern assessment is also shown, validated against ground truth. Toilet usage data are analyzed by means of a unified framework, to reliably detect statistically significant trends (linear and abrupt), as well as unexpected data points. Moreover, results of pattern clustering and anomalous pattern detection are presented, based on data coming from actual ACTIVAGE project pilots. Finally, Section 4 draws the conclusions.

## 2. Materials and Methods

As mentioned, the main concept of the ACTIVAGE DS-RER project consists of enhancing current care management practice by providing insights from user-specific “behavioral” analysis: the approach aims at creating a multi-dimensional vision of care, allowing for a more sensible personalization. To this purpose, a comprehensive framework has been devised and implemented, consisting of:

- a set of IoT home sensing devices, featuring low intrusiveness, high usability and low cost;
- a cloud infrastructure, connecting home devices to the behavioral analysis engine(s) run by the care provider;
- a set of methods allowing for extracting relevant behavioral information from raw data coming from sensors.

Besides technical functionality, it is relevant to assess the actual benefit brought to both the end-user and the care provider. This depends on the effective integration of the proposed solution into the care ecosystem, which involves a large number of different stakeholders. Hence, the framework strictly needs to be validated against real-world use cases: within the scope of the ACTIVAGE DS-RER project, a comprehensive trial procedure has been designed. This section therefore deals with the framework architecture and its components, as well as with the trial design. All technical design has been completed, and the trial is currently under way. Functional validation results are available already, based on data streams coming from pilots. Assessing evidence of clinically-relevant outcomes (based on healthcare system feedback) is among the long-term goals of the current trial and is expected at a later stage.

### 2.1. IoT Wireless Sensor Kit

The IoT wireless sensor kit is composed of several different elements, which are used to continuously monitor and extract relevant behavioral information. In order to minimize the installation burden, sensors exploit the standard Wi-Fi (IEEE 802.11 b/g/n) communication infrastructure. While most diffused home sensing networks use dedicated networks (e.g., ZigBee, Bluetooth, Z-wave, etc.), the Wi-Fi option allows for easily connected devices to the main home network (i.e., sharing common infrastructure), with no need of a dedicated home sensor hub. This makes installation straightforward and reliable. In addition, the resulting approach is highly flexible and expandable, with no need for reconfiguring the network while adding or removing devices. The ACTIVAGE DS-RER sensor kit currently includes:

- Passive InfraRed (PIR) sensors for motion detection, suitable for tracing room occupancy. Such sensors are deployed in users home by fixing them to a wall in the environment where motion needs to be captured
- Magnetic contact sensors, useful for monitoring open/close states of different objects. For example, interactions with doors, drawers and medical cabinets can be easily detected with such sensors.
- Bed occupancy sensor, useful in tracing sleeping patterns; detection of presence is achieved by a pressure-sensitive resistive pad, usually placed under the mattress. Such signal is read by the sensor module, attached to the bed frame.
- Chair occupancy sensor, to gather information on how much time and when a user sits on a chair/armchair/sofa; physical sensing technology is the same as for the bed occupancy sensor.
- Toilet presence sensor, specifically developed to keep track of daily toilet use. The sensing element is an active IR sensor with an IR illuminator and a photo-detector: this setup guarantees ranging capability and can be much more selective for close interactions detection, with respect to a PIR. Indeed, the sensor is fixed in close proximity to the toilet.

The whole sensor family was designed from scratch, taking care of both hardware and firmware design. This was necessary since no commercial WiFi sensor kit was available, providing such specific functions. In addition, interoperability and data security constraints were taken into consideration: since the ACTIVAGE project deals with information related to personal health, regulations require that the full data journey is known and the information is stored in national servers, hosted by authorized operators. The home devices kit is completed by a pill dispenser, suitable for reminding the user at medicine-taking time and for assessing the user’s compliance to the prescribed therapy. The pill dispenser was based on available hardware, and its software was updated to fit the project environment (Wi-Fi networking, cloud access).

The home infrastructure thus follows a genuine IoT approach, with sensors directly connected to the cloud, without any intermediate gateway. At any time, any element of the IoT wireless sensor kit can join the WiFi network by means of standard Wireless Protected Setup (WPS) procedure, which simplifies the device deployment. All devices are built around the same microcontroller and network processor unit, namely the CC3220 SoC (*System on Chip*) by Texas Instruments (Dallas, TX, USA): it is a Wi-Fi certified product, featuring IoT networking security, device identity and keys, optimized for low power management. The certified stack implements both IPv4 and IPv6 protocols, with industry-standard, optimized BSD sockets (both TCP and UDP), secured by SSL/TLS.

Data transmission towards the cloud is performed using the MQTT (*Message Queue Telemetry Transport*) communication protocol. MQTT is a lightweight, data-agnostic protocol, particularly suitable for IoT applications, since it it relies on a broker for exchanging data between publishers and subscribers, and it supports various levels of *Quality of Service* (QoS). In this project, all messages are sent with a QoS of 2, i.e., they are sent exactly once, with a unique reception confirmation. The Mosquitto [18] MQTT broker was chosen to receive and handle all device communications, since it is a mature technology, widely-adopted, and has an active, open-source community. After reaching the MQTT broker, a Fiware IoT agent [19] parses the messages, determines the sensor-person association, and stores the payload data into the appropriate Data Base (DB) table.

In order to guarantee data security, all traffic towards the cloud is encrypted by means of SSL/TLS protocol: certificates signed by a public Certification Authority guarantee the identification and proper authorization of devices.

To keep the installation in users’ homes less intrusive as possible, all devices are battery-powered: besides being safer in terms of electrical hazard, the absence of power cords allows unconstrained placement of sensors in the home environment. Nonetheless, battery operation comes with its own disadvantages, mainly related to the limited energy budget, the reduced capability in terms of surge current sourcing and the rate of self discharge. All these factors have a great impact on the device operating time and pose constraints on devices’ WiFi communications, which are, by far, the most power-intensive activities. In order to compensate for reduced battery current sourcing capability, the IoT wireless sensor kit relies on super-capacitors in the power section, which makes it possible to use primary alkaline cells, this reducing maintenance costs. Alkaline batteries, in fact, tend to exhibit higher ESR (*Equivalent Series Resistance*) and generally feature a more rapid performance degradation, compared to Lithium-based ones. At the very end of their discharge curve, the alkaline battery’s ESR is so high that it prevents high output current sourcing: this can seriously hinder device operation and reliability (faulty connections). The adoption of super-capacitors is common in this field and allows for offloading batteries from supplying high peaks; during normal operation, batteries will approximately supply the average current that the sensor draws, due to the higher source impedance with respect to super-capacitors: such current is relatively low, given that the sensor duty-cycle is minimal. Batteries are required to supply high currents just at the very beginning of their operation (i.e., to charge the super-capacitor up to rated voltage), when the ESR is at its minimum. This architectural pattern effectively exploits the full battery discharge curve (even in the final, high-ESR region). In the present design, two 470 mF, 4.2 V rated super-capacitor units are stacked in series, to accommodate a 4xAA alkaline battery input. This provides a low-impedance path to supply the buck regulator high amounts of transient current, required during the transmission phase. Finally, power saving capability is achieved mainly through two factors:

- Careful selection of low-power and low-quiescent current devices. For instance, FRAM (*Ferroelectric Random Access Memory*) memory is leveraged for non-volatile storage, due to their lower power consumption (compared to flash memories); low quiescent current switching regulator and analog signal conditioning ICs are selected as well, to keep sleep-mode currents as low as possible.
- Efficient scheduling of messages: instead of streaming data (e.g., movement detected by a PIR) as soon as an event occur, such information is temporarily committed to on-board non-volatile storage and sent at regular intervals (e.g., hourly). This prevents multiple WiFi connection and disconnections, effectively lowering average radio usage.

By means of such design strategy, battery lifetime well in the practical range can be achieved, in the order of several months, as detailed in Section 3.1 below.

### 2.2. System Architecture

**Figure 1:**
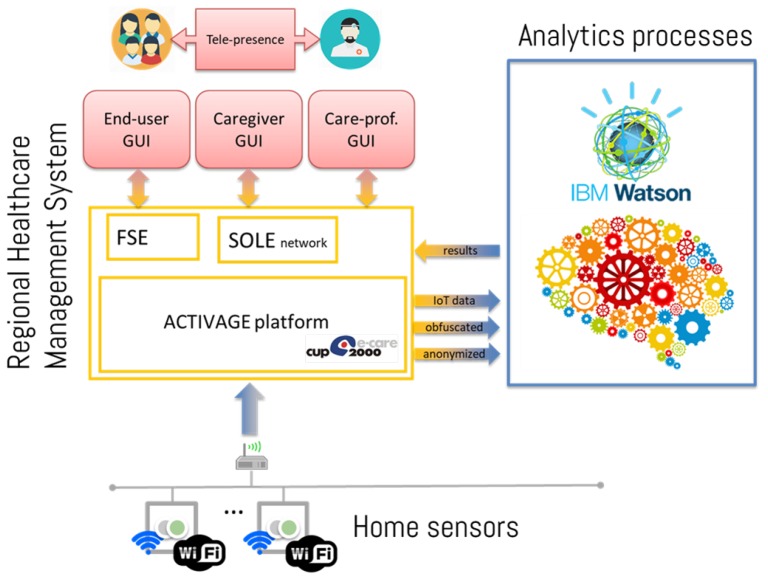
Diagram of the ACTIVAGE DS-RER system architecture, interacting with the FSE (Fascicolo Sanitario Elettronico, the patients’ interface to regional Electronics Health Record system) and the SOLE network (the interface for clinicians).

The overall system architecture is shown in Figure 1: it is built around the regional Electronic Health Records platform (progetto SOLE [20]), which is commonly used by general practitioners to manage patients’ records. Home-sensors data are sent to the platform via secure and encrypted channels, where they are stored in an anonymized fashion. To protect data privacy, the following constraints are imposed on all devices connecting to the cloud:

- sensors are only allowed to publish data and cannot retrieve any of it;
- a REST-API interface is exposed to subscribers interested in retrieving data, preventing direct interaction with the DB. In addition, such API is only exposed to clients within the project’s VPN (*Virtual Private Network*), thus providing encrypted and secure communication only to authorized subjects;
- user-generated data are pseudo-anonymized, by identifying the patterns with an alphanumeric ID and by only saving user-pilot association in a secure table, accessible only to designated and authorized parties.

Several analytics services can be defined (some of them being described in Section 2.3 below) that query anonymized data and deliver results to the same platform, where reports are securely linked to the proper subject. In particular, the analytics results are uploaded to a specific section of Electronic Health Record (EHR), managed by Local Health Authority’s, implementing the security measures listed above. Such integration allows the GP to seamlessly access the analytics insights within his usual patient management system. In addition, relevant outcomes are reported in the end-user interface as well (named “Fascicolo Sanitario Elettronico”, FSE [electronic health booklet]). In order to guarantee optimal scalability and availability, all analytics services are implemented in cloud technology, relying on the *IBM cloud* platform.

### 2.3. Data Analysis

Although suitable for unobtrusive and continuous acquisition, behavioral data produced by home sensors lacks a straightforward and absolute interpretation, especially in terms of correlation with health and wellness status: human behaviors are inherently variable, from person to person and from time to time. Hence, it is not possible to define an absolute reference to detect behavioral anomalies and features: a personalized interpretation scheme is needed. Apart from gross anomalies, relevant trends and patterns have to be evaluated in a relative fashion, i.e., by checking behavioral changes with respect to individually personalized profiles. This calls for learning capabilities in the data analytics section.

Recently, artificial intelligence techniques have been applied to smart home data, aiming at predicting user’s behavior or activity [12]. For example, the CASAS system [21] was specifically designed to perform activity of daily living (ADL) recognition from a network of home sensors: good results were achieved with Support Vector Machine (SVM) classifiers. Recognized ADLs can also represent the input to higher-level models that aim at assessing the regularity of a user’s pattern. For example, in [22], a sensor data clustering approach is adopted to obtain insights into patterns and, at the same time, to detect deviant ones. Moreover, the authors in [23] demonstrate that the extracted daily living patterns and their relative changes can be good predictors of cognitive and mobility tests performed by clinicians. In the literature, ADL discovery and classification typically rely on two factors: (i) a large number of sensors (especially PIR motion detectors), (ii) a significant corpus of user-annotated data. Both such conditions, however, are hardly compatible with real-life scenarios, due to obtrusiveness constraints.

A less demanding approach could be to use very specific sensors [24], more expressive from a semantic point of view. For example, electric appliance monitors, pressure pads, etc. can be more straightforwardly linked to specific behavioral features. Although having a narrower scope, they allow a more precise and fine-grained targeting of specific actions. Within the ACTIVAGE project environment, most IoT sensors (introduced in Section 2.1) provide action- and subject-specific information indeed. Such approach is particularly suited for persons living alone (for which continuous monitoring is especially attractive). When multiple persons populate the monitoring scene, some sensors (PIR and toilet presence sensors, most notably) cannot distinguish between different persons. Trends and anomalies can be considered anyway, but care in the interpretation is needed, since anomalies may be triggered by other users. Nonetheless, detecting such conditions in aggregated sense is still important, triggering further investigations by involved care managers. Moreover, it is worth remarking that the analyses which can be practically carried out are necessarily unsupervised: any user feedback or annotation is unfeasible or unpractical. Therefore, all analysis methodologies are focused on data interpretation (in the long term), rather than future data prediction: this is, indeed, particularly useful for care managers, who need to analyze current behaviors and detect meaningful deviations within them.

As mentioned, the DS-RER approach is applicable to many different target behaviors; however, for clearer presentation purposes, the following subsections focus on two aspects: toilet usage and bed/rest routines, which were suggested by stakeholders interviewed in co-creation sessions as important factors to be quantitatively and qualitatively monitored in post-stroke treatment.

Monitoring sleep habits of individuals after the stroke event is relevant indeed: sleep disorders, such as hypersomnia, excessive daytime sleepiness and insomnia are observed in up to 40% of individuals with chronic stroke (defined as more than six months following stroke) and 70% of those with acute stroke [25]. Proper resting is key to physical and mental health indeed [26,27,28]: sleep disturbances can lead to short-term and long-term negative patient outcomes [29], may reduce the ability to learn new motor skills [30] and increase the risk of a stroke relapse [31].

Detailed studies on sleep quality analysis are available in the literature, based on complex polysomnography setups [32,33] radio-frequency imaging [34] or video analysis [35]. Such approaches, however, do not fit well in the DS-RER approach, which focuses on low-cost, low-intrusive and self-manageable solutions instead. Similarly, monitoring of toilet usage patterns is relevant for the population at hand. Many studies in literature [36] confirm that up to 25% of acute post-stroke patients experience urinary incontinence problems even one year after the adverse event. This condition was also found positively linked with of increased morbidity, disability, and institutionalization rates in the post-stroke patient [37].

It is worth underlining that such indicators do not automatically trigger any action or therapy adjustment, but they just provide the GP or the care-manager with deeper insights about changes and anomalies in patient’s lifestyle, drawing their attention towards details which might remain unnoticed otherwise. Evaluating such information into the general framework of patient’s health is the responsibility of skilled professionals, who can assess their relevance in a personalized picture.

The DS-RER approach is general, and the methodologies detailed in the next sections can be easily adapted to many different use cases behavior; however, for conciseness’ sake, the rest of the paper will focus on bed/rest routines and toilet usage, which will serve as application examples for analytics methods explanation

#### 2.3.1. Regression Framework and Applications

It was considered that the number of daily and, possibly, nightly toilet visits should be counted and monitored, looking for meaningful behavioral changes, possibly correlated to health issues. Given the nature of the problem at hand, the natural framework for carrying out such explanatory analysis is Generalized Linear Models (GLM). In particular, the special case of Poisson regression is used, since it accounts for discrete-valued observations. Poisson Regression assume that the count data $Y_{i}$ can be modeled as independently Poisson distributed random variables, under the effect of *k* covariates $x_{i}$:

(1) $$P\left(Y_{i}=y_{i}|x_{i};\;\beta \right)=\frac{{\left(x_{i}^{T}\beta \right)}^{y_{i}}}{y_{i}!}e^{-x_{i}^{T}\beta },$$

where β is a vector of k+1 parameters (for the *k* covariates, plus a bias term) fitted on observed data. In particular, it is known that the conditional mean count, given the vector of covariates $x_{i}$ is:

(2) $$E\left[Y_{i}|x_{i};\beta \right]≜\mu_{i}=e^{x_{i}^{T}\beta }.$$

It is also known that the marginal effect of a single factor is multiplicative, i.e., a unit increment of a given covariate *j* results in a multiplication of the expected counts by a factor equal to the given parameter $\beta_{j}$. In the case at hand, at each point in time $t_{i}$, the last 30 daily counts are modeled using the Poisson regression framework, under the effect of a bias term (*baseline*) and three covariates:

- an *abrupt trend*, focusing on the most recent days (e.g., the last 5).
- an intermediate period, before the abrupt trend that allows for accounting for a past abrupt trend, without raising the baseline too much.
- a *linear trend*, to model long-term trends over the whole window.

This model is useful to reliably detect the presence of statistically significant factors, such as recent, abrupt behavior changes. For the analysis, only statistically significant factors are retained (i.e., with a *p*-value < 0.05) and used for model fitting, ensuring proper explanatory power. If, for example, a significant abrupt or linear trend is detected by the model, an alert can be triggered to automatically notify care professionals. At the same time, data points that fall too far from the predicted mean counts (e.g., outside the region that contains 95% of the distribution at that time point) can be labeled and reported as unexplained, possibly worthy of further analysis.

Of course, such regression frameworks can be easily adapted to real-valued values. For example, the *Rests Analysis Service* (RAS) aims at providing personalized periodic reports on bed usage, in order to identify significant individual changes in the short and long term. The service analyzes data collected by bed occupancy sensors and, if available, it combines them with information from other sensors, in order to confirm user movements or absence from bed, therefore reducing errors. Short bed presences, unrelated to sleep activity (as it frequently happens in real-life data), are filtered out. On the other hand, data fusion from other sensors is leveraged to confirm the end of a rest period.

Furthermore, the extracted information about factor strengths and intercepts may act as descriptive statistics in higher-level models. For example, it is possible to cluster different subjects into groups based on their features extracted with the help of such models: this can deliver further insights to care managers, which may detect similarities between care cases. Comparison between time series of different periods or different subjects is also supported by means of cosine similarity scores.

#### 2.3.2. Sensor Profiles and Applications

Another important aspect in the analysis of users’ behavior is the monitoring of temporal patterns, i.e., habits or routines. Actually, users may exhibit different habits, variable from person to person and, for the same person, from time to time. Even though explained in the following by means of the rest analysis example, this is a general topic, applicable to all data streams from the IoT sensor kit. With reference to resting habits, the necessity of modeling day-long patterns arises from the following considerations: for instance, in some cases, people rest in bed only overnight, whereas in some others daytime naps may be taken. Such routines might also shift temporally or dilate/shrink in duration. Thus, it is not possible to account for all these variabilities with just a single indicator (e.g., amount of time in bed). In order to process such information within a unified framework, *Sensor Profiles* (SP) are introduced. By breaking up a day into a suitable number of time bins (e.g., 15, 30 or 60 min intervals), SP model the expected probability of having a sensor active within each bin. It is worth remarking that the main purpose of SP analysis is to capture temporal habits rather than computing precise events’ duration. Indeed, each time bin carries the information whether the given sensor was seen sufficiently active in that time frame (either a minimum number of activations or minimum continuous active time): this allows for discarding short or non-meaningful interactions. The choice of the SP bin size plays an important role: setting bins that are too long may lead to a suppression of relevant behavioral information, whereas bins that are too short may yield noisy estimates. A bin width of 30 min was selected as good trade-off for sleep-pattern analysis. SP are then constructed as follows: for each bin, for each day, if the bed sensor is seen active for at least $t_{min}$ min, it is marked as a positive realization for that day (i.e., a value of 1 is associated with the bin); otherwise, it is marked as a negative realization (i.e., a value of 0). Thus, each time bin can be represented as a random variable $X_{j}$, whose realizations are drawn according to the following rule:

(3) $$X_{j}^{\left(i\right)}=\left\{\begin{array}{cc} 0, & \mathrm{if}\;t_{active}<t_{min},\\ 1, & \mathrm{if}\;t_{active}\geq t_{min}, \end{array}\right.$$

where $X_{j}^{\left(i\right)}$ is the *i-th* realization, corresponding to day *i*, of the *j-th* time bin, $t_{active}$ is the time the bed sensor was active (i.e., person laying in bed) on day *i* in time bin *j*. It is then straightforward to model the time bin $X_{j}$ as a Bernoulli(p) random variable: the probability parameter *p* can then be interpreted as the expected probability of finding the subject in bed during the considered time bin. Applying the principle of Maximum Likelihood Estimation (MLE), it is well known that $\hat{p}=n_{POS}/N$, where $\hat{p}$ is the estimated probability parameter, $n_{POS}$ are the number of positive realizations (i.e., days with the sensor seen active, during the considered time bin), and *N* is the total number of realizations (i.e., days). Of course, the underlying simplifying assumption is that each day is independent of the others. Confidence intervals can also be estimated, in order to quantify the $\hat{p}$ parameter uncertainty. The procedure can be repeated for each time bin $X_{j}$, with j=(1,…,24 h/binwidth), therefore modeling the probability of bed presence throughout the day.

The SP framework also lends itself well to detect behavioral pattern changes. In fact, two periods may be compared to detect statistically significant deviations in time bin estimated probabilities. For each couple of time bins $\left(X_{j},\;X_{k}\right)$, with associated probabilities $\left({\hat{p}}_{j},\;{\hat{p}}_{k}\right)$, it is possible to compare them by applying the binomial proportion statistical hypothesis testing framework. This can take the form of analytic tests, such as chi-square, or other Bayesian methods using MCMC (*Markov Chain Monte Carlo*) simulations, which can be more robust (i.e., less extreme) in reduced sample size problems. The resulting *p*-values can adjusted using the Holm–Bonferroni procedure, in order to account for multiple comparisons of all time bins.

The SP framework also allows for performing daily pattern clustering. As previously mentioned, considering a feature vector composed of daily time bins’ realizations $x^{\left(i\right)}={\left[x_{1}^{\left(i\right)},\ldots ,x_{Nbins}^{\left(i\right)}\right]}^{T}$ (where superscript *i* is referred to a single day), it is possible to perform pattern clustering by means, for example, of Agglomerative Clustering. This procedure allows for relaxing the assumption about the existence of a unique, average behavior: a person may exhibit more than one pattern. For example, a user may spend more time in bed during weekends, or may want to take a short rest in bed after lunch. This analysis may provide further insights on subjects’ habits.

Finally, within the SP framework, it is also possible to derive a *Novelty Score* (NS), to detect changes of a single day with respect to a reference period. In particular, let us suppose to have extracted a prototype pattern from said reference period, represented by a vector $\theta =\{\theta_{1},\ldots ,\theta_{Nbins}\}$ (each $\theta_{j}$ is the MLE estimate ${\hat{p}}_{j}$ of the time bin’s probability parameter). As mentioned above, for a given day *i*, let us then consider its vector of realizations $x^{\left(i\right)}={\left[x_{1}^{\left(i\right)},\ldots ,x_{Nbins}^{\left(i\right)}\right]}^{T}$; by assuming conditional independence between time bins *j*, it is possible to compute the log-likelihood of a day $x^{\left(i\right)}$, with respect to the model θ, as the sum of the log-likelihoods of each time bin realization $x_{j}^{\left(i\right)}$. The negative log-likelihood can then be taken as the NS indicator:

(4) $$NS=-\sum_{j=1}^{Nbins}\log p(x_{j}^{\left(i\right)};\theta_{j}).$$

The greater the difference of the day-vector $x^{\left(i\right)}$ with respect to the reference prototype θ, the higher the NS score is. This allows for flagging deviant days as those with a sufficiently high NS score.

### 2.4. Trial Design

As previously mentioned, the ACTIVAGE DS-RER project was designed to eventually evaluate IoT-based behavioral monitoring as a mean for improving care continuity of persons undergoing stroke recovery.

Besides technological innovation, chances of practical success of the approach strictly depend on the effective integration into current care practices. This involves acceptance from care professionals and seamless cooperation with existing care-supporting tools (e.g., Electronic Health Record management): a user-centric service design methodology was strictly followed in devising service features, directly involving General Practitioners (GP), neurologists, formal caregivers, physiotherapists and actual patients. Actual impact of ACTIVAGE service in improving care effectiveness, instead, needs to be verified through a posteriori rigorous evaluation of trial outcomes. Thus, the proposed approach was formally designed as a clinical study, adopting a strict Randomized Controlled Trial (RCT) protocol. All methodologies and evaluation procedures were submitted to the Local Health Authority’s (LHA) Ethical Committee, receiving full approval (Resolution nr. 0002775, dated 16/01/2018). Up to 200 stroke-recovery patients are being recruited, consisting of over-65 years old persons with an assessed vulnerability index from vulnerable up to moderately frail (levels 4–6 over a full range of 9). As of RCT protocol, recruited subjects are split into active and control groups, following a blind randomization process (to eliminate any selection bias). Both populations will undergo the standard protocols for stroke recovery; in addition, for the active group, such standard care practices are integrated with behavioral analysis components.

Data management is compliant with EU regulations, with details about organizational and legal issues going beyond the scope of this paper. In order to assess the impact of the ACTIVAGE solution, several performance indexes are compared among two groups, including, for instance, the re-hospitalization rate, days spent at the hospital, and complications related to co-morbidities. In addition, periodic assessment of the patient condition are carried out by means of clinically-validated questionnaires, including: Barthel index [38] for Activities of Daily Living (ADL) performance scoring, the Lawton Instrumental Activities of Daily Living Scale (IADL) [39] for assessing ability to perform task using common appliances, the Kane scale for social interaction assessment [40] and the UCLA Loneliness Scale [41]. A detailed discussion of such indicators falls beyond the scope of this technical paper: they are mentioned here to highlight that the outcome evaluation is carried out on a multi-dimensional perspective, including evaluation tools commonly used in the application context. Conforming to familiar work environment and tools should foster acceptance by the care professional indeed.

## 3. Results and Discussion

In this section, selected results are discussed, related to both the hardware and software components of the ACTIVAGE framework. In particular, power performance of IoT sensors is illustrated, as a key issue for practical and effective deployment at the actual users’ homes. Then, some analytics examples are shown (among many possible) to demonstrate how different techniques are used to provide caregivers and care professional with expressive and meaningful information, breaking down the complexity of directly dealing with raw sensor data.

### 3.1. IoT Sensor Power Profiling

Wi-Fi sensors are becoming increasingly popular, as an effective mainstreaming option for many application fields (home automation, most notably). However, the high bandwidth supported by such protocol usually comes at the cost of relatively high power consumption. Hence, in order to implement battery-powered devices in a usable fashion, special care needs to be devoted in energy budget administration. As mentioned in Section 2.1, low-power design solutions were exploited: their impact is briefly analyzed in the following.

Current consumption of IoT sensors was measured under different conditions: in fact, depending on the task being performed (such as radio communication, event logging, sleep), or on the type of sensing element (i.e., contact or IR) different power demands are needed. Table 1 summarizes such measures.

As expected, data transmission is, by far, the most power demanding activity, with up to 290 mA being sourced. To limit transmission phases, data are grouped in bursts to be transmitted at periodic intervals, as introduced in Section 2.1. Burst-based transmission greatly reduces transmission overhead, with respect to a single-event asynchronous approach: for the example, at hand, a signaling interval of 1 hour was chosen as a good trade-off between power saving issues and prompt information update. It is indeed worth remarking that no information is lost: sensor data are just temporarily stored, along with its timestamp, until the next transmission burst. From the Table 1, it is also evident that power consumption depends on the kind of sensor: as expected, more current is required to operate infrared sensors (toilet, PIR), with respect to contact-based ones, also because of the need (in the former case) of continuous polling. Therefore, choice of the polling interval impacts the overall power consumption: an period of 30 s was found to be reasonable for assessing toilet presence while allowing to enter power-saving (sleep) mode during “off” time, in which supply currents are reduced by a couple of orders of magnitude.

**Figure 2:**
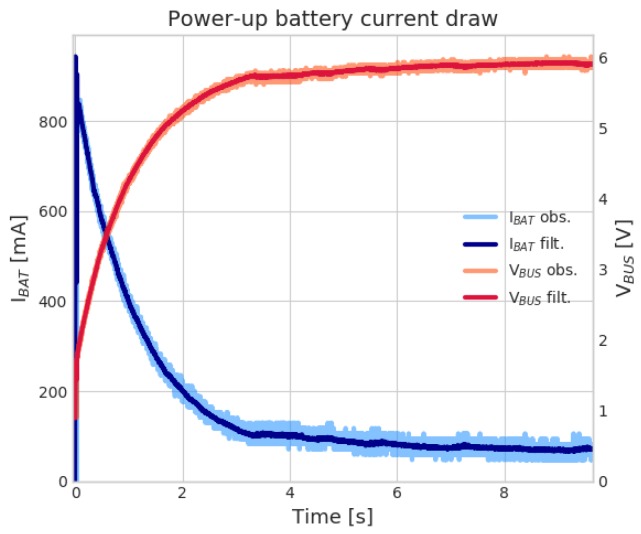
IoT sensor power-up. A large current peak is observed (blue line, left *y*-axis), corresponding to the super-capacitors charge current; meanwhile the bus voltage (red line, right *y*-axis), which is connected to the super-capacitors, ramps-up to the nominal value. After charging, the current then settles around an average of 80 mA, while the sensor scans WiFi networks for joining.

In Section 2.1, the adoption of supercapacitors is introduced to deal with momentarily current surges, not supported by aged batteries. We tested such condition by faking the higher ESR of a nearly-exhausted battery by inserting a 3.7 Ω series resistor between battery terminals and the super-capacitors. Then, we powered up the device, and left intentionally the device in the Wi-Fi network scan phase (to account for high current draw). Figure 2 shows such device power-up transient, with the system correctly turning on even though the battery performance is severely degraded. A large current peak is initially observed, followed by an exponential decay, corresponding to super-capacitors charging. Current settles around 80 mA, namely the average current drawn by the Wi-Fi network scan state (in which the device was intentionally left).

To predict actual battery lifetime, accelerated tests were conducted, by accounting for more activity (i.e., higher on/sleep duty-cycles) and more frequent data transmission: based on such test, it is estimated that, in a typical scenario, all sensors should feature a battery lifetime well over six months; the toilet sensor (which is the more power-hungry device) should run for over seven months, whereas remaining ones should approach a one-year lifetime. Of course, even better performance can be obtained by dedicated sensor-network protocols: nevertheless, the measured figures fall in the practical range, indeed; furthermore, such a relative drawback is largely compensated by advantages in terms of ease of installation and maintenance, as well as interoperability and expandability.

The recruitment of end-users and deployment of sensors at their homes is under way: at the time of writing, 18 different pilots have been installed and started to produce meaningful data (an initial latency time is needed to allow for learning). Each pilot involves (at least) three users: the elderly person, his caregiver and a care manager (or GP). Hence, about 50 users have been involved in the described pilot subset. Each pilot is equipped with the complete sensor kit described above. Apart from pill dispenser (which carry specific information, not exploited for behavioral analysis) 80 sensors are considered here, summing up to a total of 8970 sensor usage days. Within such figure, 8231 days (i.e., 91.7%) exhibited “ideal” behavior, with all scheduled transmissions properly completed. It is worth remarking that a missed transmission does not necessarily imply a data loss: data are stored in local memory and retransmitted at the following period end. The buffering capacity of the local sensor memory depends on the density of sensor data: if, for the sake of simplicity, we assume such capacity being in the order of one day of sensor data (which is a quite conservative figure indeed, with most of sensors being able to properly operate over longer network blackouts) and consider as “severe” transmission issues those exceeding such buffer capacity, we find just 17 such events (i.e., 0.19%). The cause of transmission fails does not necessarily come from sensors themselves: the whole chain needs to be accounted for. Therefore, such figure depends on a number of factors, including the home WiFi coverage, proper maintenance of devices and quality of Internet Services provision. Overall, the proposed approach (when deployed in a real-world context) exhibits a satisfactory reliability, more than sufficient for enabling the aimed continuous monitoring features. It is worth highlighting that, in order to assess the impact on clinical practice, much more factors come into play, which are not covered here: organizational issues, as well as caregiver subjective perception, will be assessed through questionnaires and focus groups at a later stage of the project.

### 3.2. Regression Framework Results

Raw sensor data require thorough processing in order to extract meaningful insights: relevant behavioral anomalies and trends need to be automatically recognized and brought to the user’s or caregiver’s attention. In addition, their relevance needs to be assessed on a relative basis, by comparing current data with learned habits. In this section, a few results are shown, for illustrative purposes: a much wider range of information is inherently available though. As stated, toilet habits are potentially relevant to health assessment: daily count data from toilet usage of real patients were analyzed by means of the Poisson regression framework introduced above. The analysis is carried out in a rolling fashion, i.e., at each day, the last 30 days of count data are used to build and fit the explanatory model: results of trend detection and anomalies are referenced to the last day and saved each time the analysis advances by one step.

**Figure 3:**
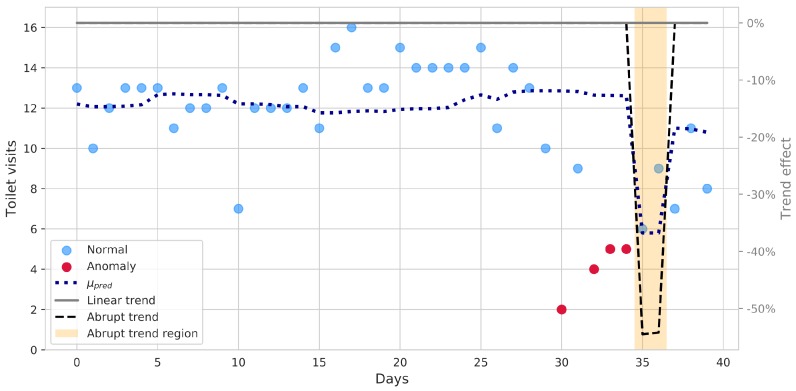
Analysis of toilet count data by a rolling Poisson regression model: blue dotted line represents the predicted mean counts. A significant abrupt trend is detected in the last days (the region is highlighted by the orange area, whereas the effect of the abrupt trend is shown by the black dashed line, along with the linear one, in solid gray). Data points not properly explained by the model are highlighted in red.

Figure 3 shows the outcomes of such daily-rolling regression analyses, applied to a real patient monitoring data. A meaningful time frame has been selected, highlighting occurrence of an actual behavioral anomaly and showing how the system accounts for it. The blue, dotted line represents the predicted mean counts, explained by the statistically significant factors, relative to the last day (day 1 in the graph accounts for the previous 30 days, not shown). For each daily step, the last day likelihood is computed, given the fitted model: if it is such that the point is outside the interval of the 95% most probable values, the point (marked in red in the graph) is labeled as *unexplained* and signaled for further potential analysis. The “outlyingness” threshold can be adjusted to make the system more reactive, if needed. To better evaluate anomalies, however, it is convenient to check for more expressive trend indicators: both “long-term” and “abrupt” trend could indicate clinically-relevant changes and are shown in figure. In the case at hand, the long-term, linear trend does not suggest any slow behavioral change, whereas some unexpected events happen at around day 30: initially, the system recognizes some abnormal days, and then evaluates them as an abrupt behavioral change (i.e., different from singularities), shown by the black dashed line. The trend is expressed as a relative increase/decrease, with respect to the baseline: statistically significant changes are highlighted by the shaded area. Such information can be exploited to properly and promptly warn the caregiver and, at the same time, to suitably update the model. Subsequent days prediction are thus accordingly adjusted, avoiding the anomaly to result in multiple detections.

### 3.3. Sensor Profiles’ Results

Similarly, the regression framework can also be successfully applied to the analysis of rests; however, for conciseness’ sake, we do not report here further examples and focus on the application of the Sensor Profile (SP) methodology introduced in Section 2.3.2. SP may provide a more comprehensive insight about user’s habits and related changes; such methodology was applied to data coming from real ACTIVAGE project monitored environments. In order to validate the approach, bed presence SP was constructed, with a time resolution of 30 min. i.e., each SP trace represents the probability of finding the subject in bed at a given time of the day.

Agglomerative Clustering was applied to the extracted sensor traces, in order to mine recurrent and characteristic patterns. A cosine similarity metric is used to compare daily traces, and the optimal number of clusters $n_{CLUS}$ is automatically selected according to two criteria:

1. in order to be considered, a cluster should have at least $n_{SAM}$ samples (in this example, $n_{SAM}=5$);
2. the parameter $n_{CLUS}$ that maximizes the average silhouette score of valid clusters (according to criterion 1) is selected.

**Figure 4:**
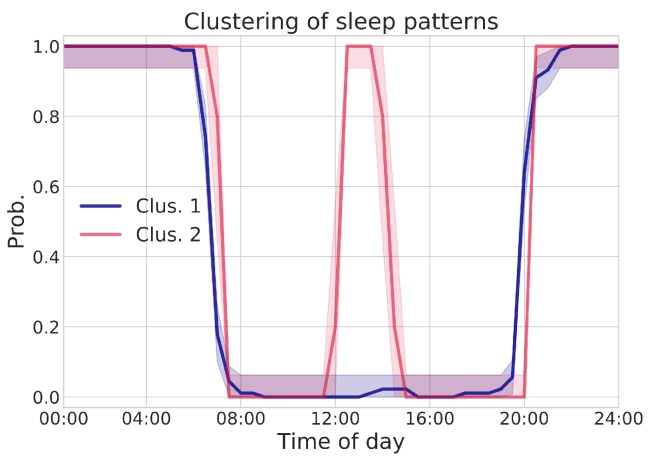
SP visualizations, resulting from data-driven pattern clustering. Average SP are plotted as solid lines, representing the probability of having the person resting in bed at each point in time for the day. Shaded area, instead, represent the uncertainty, in the form of 95% confidence intervals, in such point-wise probability estimate. The time in *x*-axis is referred to UTC, whereas the pilot timezone is UTC+1. The deviations between the two pattern clusters, from 12:30 p.m. to 2:00 p.m. (UTC) are found to be statistically significant (p<0.01).

Figure 4 shows the results of clustering operation, where a $n_{CLUS}=2$ parameter was determined from data, indicating the existence of two main clusters, corresponding to frequent behavioral patterns. Each cluster represents an SP trace as a function of time: solid lines represent the MLE estimate $\hat{p}$ (i.e., probability of bed utilization at that specific time), whereas shaded areas quantify the uncertainty of those estimates (95% confidence intervals). It is worth remarking that time, on the *x*-axis, refers to the UTC timezone: actual local time of the subject under analysis is UTC+1.

From the analysis of aggregated SP traces, two different sleep routines emerge: in both clusters, the user wakes up at about 7:00 a.m., and returns to bed at about 9:30 p.m. Just after lunchtime, instead, cluster 1 and cluster 2 differ, with cluster 2 exhibiting bed presence during the 1:30 p.m.–3:00 p.m. interval, not shown in cluster 1.

Once extracted, it is possible to compare the clusters, to check whether the visualized differences correspond to actually significant deviations, from a statistical point of view: this also helps to interpret possible behavioral shifts. Analysis is performed by adopting the binomial proportion hypothesis testing framework, allowing for comparing differences between two SP populations (i.e., clusters) in terms of expected activation probabilities of each time-of-day point. In particular, statistical significance is achieved, comparing the 1:30 p.m.–3:00 p.m. interval (p<0.01). This result is also visually confirmed in Figure 4, where confidence intervals of both clusters’ SP do not intersect and are widely spaced. Even more interesting is the fact that those deviating patterns were found in temporally close days: this shows the potential of the SP framework to detect new and significant changes in usual patterns.

The interpretation in this case is again almost trivial: the same user may have a short nap after lunch or not, depending on many circumstances. Despite such obvious meaning, this well illustrates potential of the SP modeling: since we are looking for behavioral changes (to be correlated to health or wellbeing shifts), it is quite important to recognize individual “normal” behavior to be assumed as a reference. In real life, however, a unique reference behavior does not necessarily exist, and multiple behavioral “modes” may occur, all of them to be considered “normal”. For example, the after-lunch nap option (or its absence) should not trigger any anomaly warning. The clustering technique allows for describing reference behavioral patterns in a multi-modal fashion, with multiple reference profiles automatically extracted in a completely data-driven approach. Once different behavioral modes are extracted, current behavior can be compared with all of them, and anomalies can be inferred when a profile does not match any of the identified daily prototypes.

**Figure 5:**
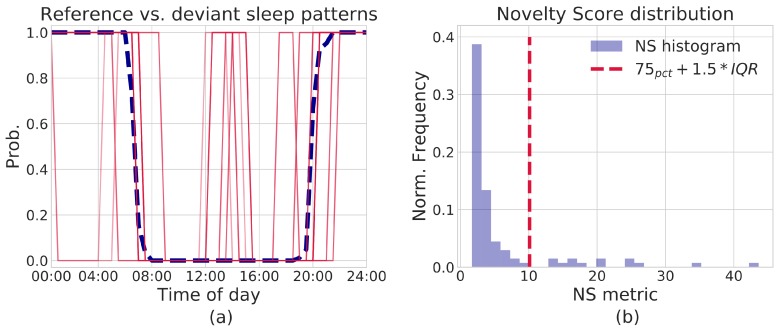
(**a**) graphical representation of “deviant” patterns, discovered with the NS score. A blue, dashed line represents the pattern taken as reference, whereas solid red lines show profiles with a high NS score, i.e., deviating from the reference; (**b**) histogram approximation of NS distribution obtained from SP traces shown in (**a**). Deviating patterns are identified by means of a simple filtering based on Inter-Quartile Range.

The notion of Novelty Score (NS) helps in evaluating such matching. The NS metric can be used to highlight deviant patterns, with respect to a given SP assumed as reference. In order to simplify the discussion, without any loss in generality, let us take the centroid of cluster 1 (${\hat{p}}_{CL1}$) in Figure 4 as reference pattern; the NS metric is then computed on all SP traces, with respect to such prototype. Deviant patterns can be highlighted by visualizing NS scores’ distribution and setting an appropriate threshold: this can be either chosen arbitrarily or automatically computed from data, by means of simple Inter-Quartile Range filter or more sophisticated solutions, including Isolation Forests or Local Outlier Factor. In this example, a simple IQR rule is sufficient:

(5) $$Daily\;trace=\left\{\begin{array}{cc} inlier, & \mathrm{if}\;NS<IQR_{Thresh},\\ outlier, & \mathrm{otherwise}, \end{array}\right.$$

where $IQR_{Thresh}$ is set to $75_{percentile}^{th}+1.5*IQR$. For the data being considered, an $IQR_{Thresh}\approx 10.1$ is derived. Figure 5a describes such an example procedure: the reference pattern is shown as a blue dashed line; in the same figure, all patterns that yield an NS score higher than $IQR_{Thresh}$ are plotted as well (red solid lines). As can be noticed, all patterns with a high NS score largely differ from the reference one. Actually, the identified deviant patterns are those from cluster 2, together with a couple too far from both clusters (part of a cluster which was under-represented according to Criterion 1 above). This *outlyingness* is more evident in Figure 5b, which shows a histogram representation of the computed NS scores, along with the determined $IQR_{Thresh}$. It is clear that the NS score neatly allows separation of deviant patterns from those very similar to the reference one of Figure 5a. Thus, the presented methodology can be effectively used to detect anomalous, unusual behavioral patterns in user data, possibly reflecting some change in user’s wellbeing. It is worth stressing that, in this case too, the user or his caregiver just receive synthesis information, with the system automatically selecting potentially relevant details and thus relieving the user/caregiver himself from continuous evaluation of sensor data streams.

## 4. Conclusions

The ACTIVAGE project, framed in the Horizon 2020 initiative, focuses on IoT-enabled Active and Healthy ageing. Within ACTIVAGE, the RER deployment site aims at improving continuity of care for older persons, suffering from stroke aftereffects. The DS-RER interdisciplinary approach combines clinical practice (general practitioners and the Local Health Authority are involved as project partners) and ICT technology, aiming at creating new services and paradigms for complementing traditional (telemedicine) practice. A complete IoT architecture has been implemented, from distributed sensing based on Wi-Fi home sensors to cloud-enabled analytics. Design and engineering of the IoT wireless kit was described, with emphasis on low-power design techniques. Advanced methodologies were introduced to recognize anomalies and meaningful trends from the analysis of raw data collected from such sensors: this provides caregivers and care professionals with concise and expressive information, suitable for supporting the patient’s care flow in a straightforward fashion.

In particular, an IoT sensor kit is installed in the pilot houses, aiming at assessing main habits and routines in their own daily living setting. Each sensor is battery operated and uses a standard, home Wi-Fi connection for securely logging data in a protected cloud environment. The approach is less intrusive and demanding than “dedicated” wireless sensor network technologies, at the same time fully complying with most recent regulations in terms of data protection and privacy. Much effort was spent in reducing the need for home system maintenance, to make the technology fully manageable by the end user himself or by his caregivers. Careful hardware design procedures have been adopted, aiming at maximizing battery lifetime. Message scheduling and careful design of the data communication protocol allow for effective exploitation of low power modes, whereas the adoption of super-capacitors in the power supply allows for using the full battery discharge curve, regardless of degraded current sourcing capability. With an average stand-by current as low as 12 μA, battery lifetimes in the 6–12 months range are to be expected, as suggested by accelerated lab tests.

The cloud analytics architecture supporting behavioral analysis is described. From such analysis, anomalies and meaningful trends are automatically assessed in an unsupervised fashion. Validation has been carried out both in a lab environment (with healthy subjects) and in real pilot settings. Results can be straightforwardly fed to the regional Electronic Health Records system, and submitted to general practitioners’ attention by means of the regional record information system. The whole data journey fully complies with most recent privacy and security regulations.

Several methods for analyzing different user data were introduced, taking toilet usage and rest behaviors as meaningful examples. In particular, in the former case, a regression framework was introduced to mine and detect statistically significant trends (both long-term and abrupt ones), while at the same time labeling unusual, deviant observation. The Sensor Profile framework was then introduced, which allows for unifying analysis methodologies of arbitrary sensor traces. SP framework was applied on rest patterns measured by a Wi-Fi bed occupancy sensor in a real-life setting: applying Agglomerative Clustering, multi-modal habits assessment was carried out. This allows for detecting anomalies with respect to a set of personalized usual behaviors, instead of just a unique, averaged one. The capability of the SP framework to detect new patterns, effectively discriminating between actual emerging trends and simple statistical fluctuations, has been discussed. Based on the SP framework, a Novelty Score metric was introduced and demonstrated to provide reliable anomaly detection for bed pattern data.

The system is currently being deployed and tested over several tens of users’ homes, with the aim of both technical validation and assessment of its impact on care practices: to this purpose, a Randomized Control Trial experiment is being carried out (referring to the use case of stroke recovery) within the framework of the ACTIVAGE H2020 project. By conjugating technical architecture design and service conception in a truly interdisciplinary approach, the DS-RER solution, beyond the necessarily limited scope of the actual pilot tests, will allow for highlighting potentials of IoT technologies on the improvement and sustainability of healthcare services.

## Figures and Tables

**Table 1:** Device current consumption under different conditions

Condition	Current Consumption
WiFi Receive and network scan	80 mA
WiFi Transmit	290 mA (peak)
Wakeup and event logging:	
- bed, chair, contact	2 mA
- PIR, toilet	14 mA
Sleep (power save)	12 μA
